# Mediating effect of technostress on the relationship between artificial intelligence literacy and attitude toward digital technology among health professions students: a structural equation modeling approach

**DOI:** 10.12701/jyms.2026.43.7

**Published:** 2025-12-30

**Authors:** Jin Young Lee, Yul Ha Min, Jun Yim, Kwi Hwa Park, So Jung Yune

**Affiliations:** 1 Center of Medical Education Innovation, Pusan National University School of Medicine, Yangsan, Korea; 2 College of Nursing, Kangwon National University, Chuncheon, Korea; 3 Division of Medical Science, Inha University College of Medicine, Incheon, Korea; 4 Department of Medical Education, Gachon University College of Medicine, Incheon, Korea; 5 Department of Medical Education, Pusan National University School of Medicine, Yangsan, Korea

**Keywords:** Artificial intelligence, Attitude to computers, Mediation analysis, Psychological stress

## Abstract

**Background:**

This study aimed to examine the effect of artificial intelligence (AI) literacy on attitudes toward digital technology and the mediating effect of technostress on this relationship among health professions students.

**Methods:**

An online survey was conducted from May to October 2025 with 1,314 students enrolled in medical schools, nursing schools, dental schools, and graduate schools of dentistry nationwide. Structural equation modeling and bootstrapping analyses were performed.

**Results:**

The analysis revealed that AI literacy significantly reduced technostress and enhanced attitudes toward digital technology. Technostress also had a negative effect on attitudes toward digital technology, and a partial mediating effect was identified in the relationship between AI literacy and attitudes toward digital technology. In other words, higher levels of AI literacy were associated with lower technostress, which, in turn, led to more positive attitudes toward digital technology. Multigroup analysis further showed that the effect of AI literacy on technostress differed across majors, being significant for medical and nursing students, but not for dental students.

**Conclusion:**

This study confirmed that improving AI literacy reduces technology-related stress and promotes positive attitudes toward digital technology. These findings suggest the need for AI and digital technology education designs that consider the psychological factors of learners in medical education. Furthermore, the observed group differences suggest that AI literacy may function differently depending on discipline-specific technological and educational contexts.

## Introduction

With the rapid expansion of artificial intelligence (AI) applications in the healthcare sector, AI-based learning tools and clinical decision support systems are being increasingly adopted in medical education settings [1]. AI technologies in healthcare contribute to enhanced diagnostic accuracy, optimized treatment planning, and improved accessibility, underscoring the growing need for healthcare professionals to acquire a strong understanding of AI and the competencies required to use it effectively [2]. However, accelerated integration of new technologies poses significant challenges for learners. In particular, students in medical disciplines must simultaneously master traditional biomedical knowledge while engaging with emerging digital technologies, thereby creating a dual burden of adaptation.

In this context, AI literacy has gained attention as a core competency for medical students. AI literacy encompasses the ability to understand AI technologies, critically evaluate their use, ethically reason their implications, and integrate them appropriately into clinical and educational settings to ensure their safe and effective application [3]. Previous studies have shown that healthcare professionals with higher levels of AI literacy are better able to utilize AI-based decision-making systems, recognize their limitations, and make patient-centered and ethically sound judgments [4]. Attitudes toward digital technology, defined as an individual’s positive or negative evaluations and beliefs regarding the use of digital technologies, represent a key psychological factor that influences the individual’s willingness to adopt and meaningfully engage with such tools [5]. Evidence suggests that students with higher AI literacy tend to develop more positive attitudes toward digital technologies [6].

Despite the potential benefits of AI integration, adapting to new technological environments can place a considerable psychological strain on learners. Technostress refers to the psychological and cognitive burden experienced when individuals struggle to adapt to novel technologies [7,8], and encompasses factors such as technological overload, complexity, and anxiety [9]. Health professions students may be particularly vulnerable to technostress given the dual demands of learning vast amounts of medical knowledge while also acquiring proficiency in a variety of digital tools, including AI systems [10]. Prior research indicates that lower levels of AI literacy are associated with greater perceived technological complexity and anxiety, which in turn elevates technostress levels [10,11]. Technostress has been shown to contribute to negative perceptions of technology and reduce the intention to adopt or continue using digital tools. Meta-analysis further suggests that technostress negatively affects both psychological (e.g., satisfaction, burnout) and behavioral (e.g., technology use intentions and sustained use) outcomes [9]. These psychological and behavioral responses play a central role in shaping attitudes toward digital technologies [5,11]. In Korea, recent research has started exploring AI education for health professions students, showing that structured AI-based teaching methods can effectively improve AI understanding and preparedness of students in clinical contexts [12]. Although the incorporation of AI in medical education is accelerating, current evidence suggests that AI literacy among health professions students remains insufficient. A recent systematic review reported that only 44% of medical, dental, and nursing students possessed adequate AI knowledge, highlighting the need for more effective educational interventions [13]. Furthermore, few studies have examined the structural relationships among AI literacy, technostress, and digital technology attitudes in the context of medical education, and empirical studies investigating the mediating role of technostress among these variables are scarce.

**Fig. 1.**
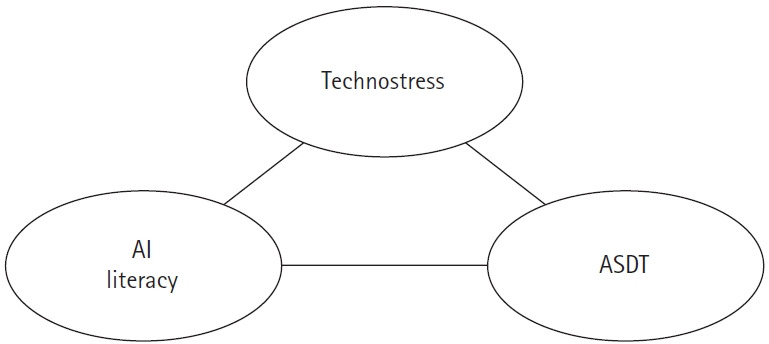
Research model of artificial intelligence (AI) literacy, technostress, and attitude scale for digital technology (ASDT).

Accordingly, using a structural equation modeling (SEM) approach, this study aimed to examine the effects of AI literacy on digital technology attitudes among health professions students, with a specific focus on the mediating role of technostress. The hypothesized relationships among AI literacy, technostress, and attitude toward digital technology are illustrated in Fig. 1. Specifically, this study investigates (1) the direct effect of AI literacy on technostress, (2) the direct effect of AI literacy on digital technology attitudes, (3) the direct effect of technostress on digital technology attitudes, (4) the indirect (mediated) effect of AI literacy on digital technology attitudes through technostress, and (5) whether the structural paths in the research model differ across academic majors. The findings are expected to provide empirical support for the importance of AI literacy education in medical training and contribute to the development of effective educational strategies that incorporate technostress management.

## Methods

**Ethics statement:** The survey procedures were conducted in accordance with ethical standards after obtaining approval from the Institutional Review Board (IRB) of Gachon University (IRB No: 1044396-202501-HR-006-01). On the first page of the online questionnaire, all participants were informed about the purpose of the study, the voluntary nature of participation, and data confidentiality, and only those who provided informed consent completed the survey.

### 1. Participants

The study participants were undergraduate and graduate students enrolled in medical schools, nursing schools, dental schools, and graduate schools of dentistry nationwide. Data were collected through online surveys conducted between May 2025 and October 2025. For dentistry and nursing students, survey links were distributed through the cooperation of the Korean Association of Dental Students and the Next Generation Nursing Leader Association of the Korean Nurses Association, respectively, with voluntary participation encouraged. For health professions students, six medical schools in Seoul, Gyeonggi, Busan, Gyeongsang, and Jeolla regions were selected using convenience sampling to ensure geographic representation, and surveys were administered through each institution.

Although the survey was administered anonymously, potential duplicate responses were controlled by enabling the Google Forms setting, which restricted participants to a single submission per account. This procedure effectively prevented multiple entries from the same respondent. Of the 1,335 respondents, 21 cases involving response withdrawal, insincere responses, or missing data were excluded, resulting in a final sample of 1,314 participants for analysis.

The final sample comprised 316 medical students (24.0%), 682 nursing students (51.9%), and 316 dental students (24.0%). There were 434 male students (33.0%), 852 female students (64.8%), and 28 nonrespondents (2.1%).

### 2. Instruments

The Meta AI Literacy Scale, developed by Carolus et al. [14], measures competencies required in the AI era, including understanding, utilization, and ethical judgment. The main subcategories include Use & Apply, Understand, Detect, Ethics, and Create, along with meta-competencies such as learning self-efficacy and self-regulation. While the original instrument consists of 34 items, this study used 28 of them (excluding the self-regulation domain), measured on a 5-point Likert scale. The self-regulation domain was excluded because it reflects general learning management skills rather than AI-specific competencies and therefore, did not align with the analytical focus of this study. The reliability in this study was excellent (Cronbach’s α, 0.946).

The Technostress Scale, based on the model of Tarafdar et al. [15], was designed to assess the stress factors arising from technology use. The instrument comprises 21 items across five subcategories: techno-overload (five items), techno-invasion (four items), techno-complexity (three items), techno-insecurity (five items), and techno-uncertainty (four items), measured on a 5-point Likert scale. For the present study, the scale was translated into Korean through translation and back-translation conducted by three medical education experts to ensure its linguistic and cultural appropriateness. Reliability analysis indicated excellent internal consistency (Cronbach’s α, 0.947).

The Attitude Scale for Digital Technology (ASDT) developed by Cabı [16] was also used. From the original 39 items, only 10 corresponding to the “competence” factor were extracted and measured on a 5-point Likert scale. This subscale measures individuals’ self-perception and confidence regarding their ability to understand and utilize digital technology and solve problems. This study specifically assessed the level of self-perception of digital technology utilization competence and used only the “competence” subdimension of ASDT because it shows the highest theoretical relevance to the research model. Competence directly reflects perceived digital capability, allowing for a clearer examination of its relationship with AI literacy. The reliability of the competence subscale was excellent (Cronbach’s α, 0.919).

### 3. Data analysis

Data were analyzed using the following procedures. First, descriptive statistics for the AI literacy, technostress, and digital technology attitude competence variables were calculated, and normality was verified by examining skewness and kurtosis values. Second, correlation analysis was performed to identify relationships among the main variables. Third, SEM analysis was conducted to examine the structural causal relationships among AI literacy, technostress, and digital technology attitude competence. Fourth, the significance of the mediating effect of technostress was verified using the bootstrapping technique with 1,000 iterations and a 95% confidence interval. Finally, a multigroup SEM analysis was conducted to examine the differences in structural paths across academic majors (medicine, nursing, and dentistry). Measurement invariance was first assessed, followed by testing group differences in structural path coefficients by imposing equality constraints on each path. All data analyses were performed using IBM SPSS ver. 29.0 and AMOS ver. 29.0 (IBM Corp., Armonk, NY, USA).

## Results

### 1. Descriptive statistics and correlation analysis

The descriptive statistics of the main variables used in this study are presented in Table 1. The mean AI literacy score was 3.763 (standard deviation [SD], 0.630), indicating a relatively high level. Technostress had a mean value of 2.794 (SD, 0.833), reflecting a comparatively low level. The mean ASDT score was 3.811 (SD, 0.713), suggesting a generally positive attitude. Additionally, the absolute values of skewness and kurtosis for all variables were below 2, confirming that the normality assumption was satisfied [17,18].

The correlation analysis indicated significant associations among AI literacy, technostress, and ASDT. AI literacy showed a significant negative correlation with technostress (r=–0.274, *p*<0.001) and a significant positive correlation with ASDT (r=0.718, *p*<0.001). Additionally, technostress showed a significant negative correlation with ASDT (r=–0.300, *p*<0.001). Although the correlation between AI literacy and ASDT was relatively high (r=0.718), an examination of multicollinearity showed that AI literacy had a tolerance of 0.925 and a variance inflation factor (VIF) of 1.081. Technostress also showed a tolerance of 0.925 and a VIF of 1.081. These values are within the acceptable criteria for multicollinearity (VIF <5 or <10), indicating that multicollinearity is not a concern in the SEM.

### 2. Verification of research model

The goodness-of-fit indices for the research model are presented below. Examining the goodness-of-fit of the research model, χ²=1,622.697 (degrees of freedom [df]=203, *p*<0.001), χ²/df=7.994, the Tucker-Lewis index (TLI) was 0.915, the comparative fit index (CFI) was 0.925, and the root mean square error of approximation (RMSEA) was 0.073. Generally, when χ²/df is ≤8, the TLI and CFI are ≥0.90, and RMSEA is ≤0.08, the level is considered acceptable [18,19]. Accordingly, the structural model in this study showed an overall good fit.

The results of estimating path coefficients of the research model are presented in Table 2. The paths from AI literacy to technostress and technostress to ASDT were significantly negative (β=–0.342, *p*<0.001 and β=–0.118, *p*<0.001, respectively). By contrast, the path from AI literacy to ASDT showed a significantly positive effect (β=0.748, *p*<0.001), indicating that the higher the AI literacy, the more positive the ASDT value. Additionally, the standardized factor loadings (β) for measurement indicators of each latent variable were all ≥0.60, confirming that all items reflected their corresponding factors well, which was statistically significant (*p*<0.001).

### 3. Mediating effect verification

The results of the mediation analysis examining the role of technostress in the relationship between AI literacy and ASDT are presented in Table 3. Bootstrapping was verified with a 95% confidence interval through 1,000 repeated extractions. Since the lower limit of the indirect effect was 0.036 and the upper limit was 0.095, not including 0, the mediating effect was found to be significant (*p*<0.01). Examining the effects by path, the direct effect of AI literacy on ASDT was 1.174, the indirect effect was 0.063, and the total effect was 1.237. As AI literacy increases, technostress decreases, and lower technostress levels increase ASDT, showing that technostress has a negative partial mediating effect.

**Fig. 2.**
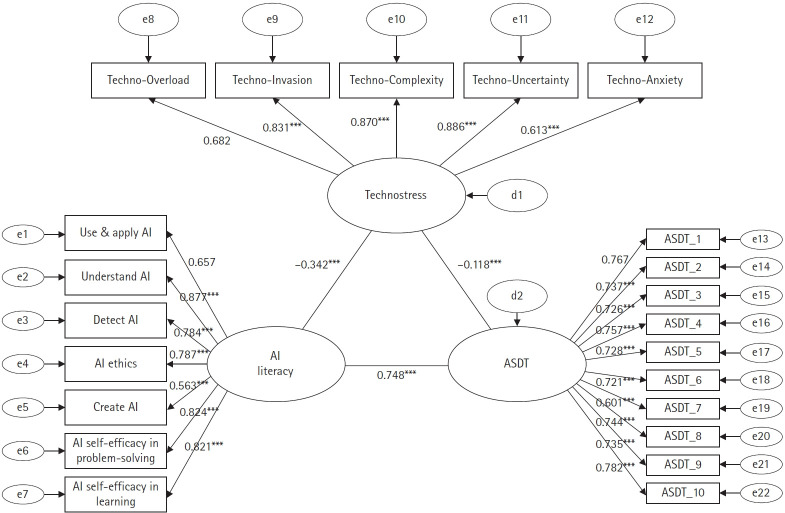
Structural path model of latent variables related to artificial intelligence (AI) literacy, technostress, and attitude scale for digital technology (ASDT). ***p<0.001.

The final path model among the latent variables established in this study is shown in Fig. 2.

### 4. Multigroup structural model evaluation and path invariance analysis

A multigroup confirmatory factor analysis was conducted to examine whether the measurement structure was equivalent across groups. Measurement invariance was evaluated following the criteria proposed by Cheung and Rensvold [19], in which a change in CFI (ΔCFI) of ≤0.010 indicates no substantial difference between models. A comparison of the configural invariance model (χ²=2,150.756; df=609; CFI, 0.920; RMSEA, 0.044) and the metric invariance model (χ²=2,329.879; df=647; CFI, 0.913; RMSEA, 0.045) showed that the ΔCFI was –0.007, which met the recommended threshold. Therefore, measurement invariance across groups was established.

Based on the confirmed measurement invariance, a multigroup SEM was performed to examine whether the structural paths differed across academic majors. The baseline structural model, in which all factor loadings were constrained to be equal across groups, demonstrated an acceptable level of model fit (χ²=2,329.897; *p*<0.001; TLI, 0.907; CFI, 0.913; RMSEA, 0.045). The path estimates for each group are presented in Table 4.

An examination of standardized path coefficients revealed that the effect of AI literacy on technostress was significantly negative for medical students (β=–0.390) and nursing students (β=–0.456), whereas the effect was not significant for dental students. Similarly, the effect of technostress on ASDT was significant in the medical (β=–0.187) and nursing (β=–0.120) groups but not in the dental group. In contrast, direct effect of AI literacy on attitude toward digital technology was significantly positive in all groups, including medical students (β=0.714), nursing students (β=0.793), and dental students (β=0.651).

A comparison of the baseline model and models with equality constraints on individual structural paths indicated that only the path from AI literacy to technostress differed significantly across groups (Δχ²=54.568, *p*<0.001). No significant differences were found for the paths from technostress to ASDT (Δχ²=4.668) and from AI literacy to ASDT (Δχ²=0.633). When equality constraints were imposed simultaneously on all three structural paths, the constrained model showed a significant difference from the baseline model (Δχ²=60.957, *p*<0.001), indicating that the overall structural model differed across groups.

## Discussion

This study investigated the relationships between AI literacy, technostress, and digital technology attitudes among students enrolled in medical-related programs, with an emphasis on the mediating role of technostress in the association between AI literacy and digital technology attitudes. After examining the correlations among the variables, SEM was employed to empirically validate the proposed mediation model.

The structural path model demonstrated acceptable overall fit (χ²/df=7.994; TLI, 0.915; CFI, 0.925; RMSEA, 0.073), meeting the criteria suggested by Bentler [20] as well as Browne and Cudeck [21]. All three hypothesized pathways were statistically significant.

First, AI literacy was found to have a significant negative effect on technostress (β=–0.342, *p*<0.001). This indicates that higher levels of AI literacy are associated with reduced psychological strain during technology use, suggesting that AI literacy is a protective factor against technostress. This finding is consistent with that of recent research in the context of medical education. Kimiafar et al. [22] reported that inadequate preparedness and limited exposure to AI heightened AI-related anxiety, while structured literacy programs effectively enhanced AI competencies among healthcare learners. Similarly, a study involving Turkish medical students found that greater AI readiness was associated with lower levels of AI anxiety [23]. Additional evidence indicates that digital competence mitigates perceived stress and burnout in remote learning environments [24]. Collectively, these studies support the interpretation that digital literacy functions as a psychological resource that buffers technology-related stress in demanding academic and clinical environments characteristic of medical education.

Second, AI literacy exerted a strong positive effect on digital technology attitude (β=0.748, *p*<0.001). This suggests that the more students understand and feel capable of using AI, the more positively they evaluate digital technologies. This was the strongest direct effect of the model. These findings align with the foundational assumptions of the Technology Acceptance Model, in which perceived competence and control over technology shape positive technology-related attitudes. Recent studies have supported these findings. Si [12] found that AI literacy was strongly associated with favorable AI attitudes and intentions to use AI in clinical practice among Korean healthcare students. Similarly, Laupichler et al. [25] demonstrated that higher levels of AI literacy among German medical students predicted more positive attitudes toward AI and reduced AI-related fear. Moreover, Cho and Seo [26] confirmed that acceptance attitudes mediated the relationship between AI perceptions and intentions to use AI among Korean nursing students. Subaveerapandiyan et al. [27] emphasized that AI literacy is essential for fostering positive attitudes toward the integration of AI into future clinical environments [27]. Collectively, these studies indicate that AI literacy education plays a central and ongoing role in cultivating favorable attitudes toward the adoption and meaningful use of AI in healthcare.

Third, technostress had a significant negative effect on digital technology attitudes (β=–0.118, *p*<0.001), indicating that psychological strain arising from information overload, system complexity, or technological uncertainty may lead to unfavorable evaluations of digital technologies. This finding is consistent with the theoretical frameworks that posit that technostress contributes to technology avoidance and negative user perception. Tarafdar et al. [28] empirically demonstrated that technostress creators increase psychological strain and ultimately undermine user satisfaction and performance. Similar patterns have been documented in education and healthcare environments. Mushtaque et al. [29] reported that technostress reduced the intentions of Pakistani medical students to use online learning during the coronavirus disease 2019 pandemic, while Liu et al. [30] observed that technostress among physicians contributed to negative affect and resistance toward mobile electronic medical record systems. Elevated technostress levels among healthcare practitioners have also been widely reported [31]. Together, these findings highlight technostress as a significant psychological barrier to forming positive attitudes toward technology.

Fourth, the mediating effect of technostress on the relationship between AI literacy and digital technology attitudes was partially supported. This indicates that AI literacy influences digital technology attitudes both directly and indirectly through stress-reducing effects. This mediating mechanism is consistent with prior research on medical and health professions education. Ibrahim et al. found that digital literacy partially mediated the relationship between emotional intelligence and academic stress [32], while Vermisli et al. [33] reported that perceived stress mediated the effect of digital literacy on satisfaction with remote education among nursing students. In addition, Ayed et al. [34] showed that AI-related anxiety significantly predicted attitudes toward AI among nursing students, highlighting the role of emotional factors in shaping technology-related attitudes. Collectively, these findings support the interpretation that psychological burdens, such as stress and anxiety, play a meaningful role in shaping attitudes toward digital technologies. These results underscore the need for AI literacy education to encompass not only technical skill development, but also strategies aimed at reducing psychological stress and promoting positive emotional engagement with technology.

Finally, the structural model proposed in this study demonstrated stable applicability for both medical and nursing students, but not for technostress-related pathways among dental students. Specifically, while the direct effect of AI literacy on ASDTs remained consistently strong across all disciplines, technostress-related pathways operated differently depending on the academic major. Notably, AI literacy did not significantly influence technostress among dental students but served as a significant factor in reducing technostress among medical and nursing students.

These findings can be explained by differences in professional identity formation across disciplines. In medicine and nursing, AI and digital technologies are increasingly integrated as core professional competencies, and AI literacy is a critical component of professional self-efficacy [6]. In particular, nurses are more frequently exposed to digital tools, and the use of complex digital systems in hospital settings is essential, making AI literacy a practical buffer against technostress [35]. In contrast, the professional identity of dentistry is centered on manual dexterity [36,37], aesthetic judgment [38,39], and patient-centered interaction [40,41], with AI being perceived primarily as an “auxiliary tool” [42]. Consequently, even when dental students possess high levels of AI literacy, this competency does not translate into enhanced professional self-efficacy, resulting in no observable effect of technostress reduction.

These findings underscore the need for discipline-specific AI education strategies. For medical and nursing students, educational interventions should integrate both technology stress management and AI competency development [35]. By contrast, for dental students, a more practical approach focusing on AI utilization skills rather than stress management may be more effective [42]. Such discipline-tailored approaches can support all healthcare students in acquiring competencies appropriate for the AI era, while respecting the unique professional characteristics of each field.

Based on these findings, several practical implications for medical education can be identified. First, the strong positive influence of AI literacy on digital technology attitudes highlights the need to integrate structured AI literacy education within the curriculum. Such programs should not only introduce foundational concepts of AI but also include practical experiences that enable students to engage with clinical AI tools, thereby increasing their confidence and competence in technology use.

Second, the negative impact of technostress on attitudes toward digital technology suggests the importance of incorporating strategies aimed at reducing the technical and emotional burdens associated with technology use. Reducing the complexity of digital learning environments, providing adequate orientation and ongoing support when new technologies are introduced, and offering reflective or peer-supported learning opportunities may help students manage their anxiety and stress more effectively. These approaches can ultimately facilitate a more positive engagement with AI and digital technologies in medical training.

Using SEM, this study empirically examined the structural relationships among AI literacy, technostress, and digital technology attitudes among students enrolled in medical-related programs. The findings revealed that AI literacy directly enhances digital technology attitudes and exerts an indirect positive effect by reducing technostress. However, the multigroup analysis showed that, while AI literacy significantly reduced technostress among medical and nursing students, this pathway was not significant among dental students.

These outcomes have important implications for medical education. AI literacy education must strengthen conceptual understanding and technical competence, while incorporating technostress management strategies. Medical and nursing students require integrated approaches, including stress management, whereas dental students require different strategies that emphasize practical AI utilization. Such discipline-tailored approaches can effectively prepare health care professionals for AI-enabled clinical environments.

Despite its meaningful contributions, this study had several limitations. First, the use of self-report questionnaires may have introduced social desirability bias or subjective interpretations by the respondents. Second, students from medical, dental, and nursing programs were aggregated into a single “medical-related student” group. Given the distinct curricular structures, clinical exposure levels, and AI integration experience across these disciplines, important discipline-specific characteristics may not have been fully captured. Third, different sampling methods were used for each major. Medical students were recruited through convenience sampling from six institutions, while nursing and dental students were recruited through national professional associations. These differences in sampling procedures may have introduced heterogeneity into the sample and affected the representativeness of each group, potentially influencing the comparisons across majors. Therefore, the findings of this study should be interpreted with caution considering the potential bias arising from these sampling differences. Fourth, as noted earlier, this study used only the “competence” subdimension of ASDT, which represents a limitation. This measurement reflects perceived digital competence rather than the full spectrum of attitudes encompassed by the ASDT. Therefore, readers should interpret the findings with this methodological constraint in mind.

Future research should address these limitations. Mixed methods designs incorporating behavioral measures, in-depth interviews, and observational approaches could enhance the validity and robustness of the findings by complementing self-report data. Additionally, comparative analyses across medical, dental, and nursing programs would help to identify discipline-specific needs and contextual differences, providing a foundation for developing tailored AI literacy education strategies aligned with the distinct requirements of each health profession.

## Figures and Tables

**Table 1.** Descriptive statistics of study variables (n=1,314)

Variable	Range	Mean±SD	Skewness	Kurtosis
AI literacy	1–5	3.763±0.630	–0.274	–0.123
Technostress	1–5	2.794±0.833	0.122	–0.707
Attitude scale for digital technology	1–5	3.811±0.713	–0.586	–0.066

SD, standard deviation; AI, artificial intelligence.

**Table 2.** Path coefficients of the structural model

Predictor	Outcome	B	β	SE	CR	*p-*value
AI literacy	Technostress	–0.535	–0.342	0.051	–10.566	<0.001
Technostress	ASDT	–0.118	–0.118	0.022	–5.265	<0.001
AI literacy	ASDT	1.174	0.748	0.057	20.459	<0.001
AI literacy	Use & apply AI	1	0.657			
	AI understand	1.411	0.877	0.052	27.197	<0.001
	Detect AI	1.447	0.784	0.058	24.883	<0.001
	AI ethics	1.352	0.787	0.054	24.958	<0.001
	Create AI	1.658	0.563	0.106	15.593	<0.001
	AI self-efficacy in problem-solving	1.549	0.824	0.060	25.903	<0.001
	AI self-efficacy in learning	1.61	0.821	0.062	25.840	<0.001
Technostress	Techno-overload	1	0.682			
	Techno-invasion	1.192	0.831	0.038	31.761	<0.001
	Techno-complexity	1.276	0.870	0.046	27.539	<0.001
	Techno-uncertainty	5.331	0.886	0.191	27.847	<0.001
	Techno-anxiety	0.893	0.613	0.034	26.394	<0.001
ASDT	ASDT_1	1	0.767			
	ASDT_2	1.058	0.737	0.038	27.929	<0.001
	ASDT_3	1.019	0.726	0.037	27.453	<0.001
	ASDT_4	0.996	0.757	0.035	28.806	<0.001
	ASDT_5	0.921	0.728	0.033	27.524	<0.001
	ASDT_6	1.156	0.721	0.042	27.208	<0.001
	ASDT_7	0.752	0.601	0.034	22.140	<0.001
	ASDT_8	1.035	0.744	0.037	28.224	<0.001
	ASDT_9	1.080	0.735	0.039	27.852	<0.001
	ASDT_10	1.067	0.782	0.036	29.933	<0.001

B, unstandardized regression coefficient; β, standardized regression coefficient; SE, standard error; CR, critical ratio; AI, artificial intelligence; ASDT attitude scale for digital technology.

**Table 3.** Effects of AI literacy on ASDT mediated by technostress

Predictor	Mediator	Outcome	Direct effect	Indirect effect (95% CI)	Total effect
AI literacy	Technostress	ASDT	1.174	0.063 (0.036–0.095)	1.237

AI, artificial intelligence; ASDT, attitude scale for digital technology; CI, confidence interval for the indirect effect.

**Table 4.** Comparison between the baseline model and equality-constrained models across groups

Predictor	Outcome	Medical students		Nursing students		Dental students	
		B	β	B	β	B	β
AI literacy	Technostress	–0.534^a)^	–0.390	–0.671^a)^	–0.456	0.140	0.097
Technostress	ASDT	–0.212^a)^	–0.187	–0.120^a)^	–0.120	–0.044	–0.037
AI literacy	ASDT	1.107^a)^	0.714	1.168^a)^	0.793	1.124^a)^	0.651

AI, artificial intelligence; ASDT, attitude scale for digital technology.

a) *p*<0.001.

## References

[1] Alowais SA, Alghamdi SS, Alsuhebany N, Alqahtani T, Alshaya AI, Almohareb SN (2023). Revolutionizing healthcare: the role of artificial intelligence in clinical practice. BMC Med Educ.

[2] Han ER, Yeo S, Kim MJ, Lee YH, Park KH, Roh H (2019). Medical education trends for future physicians in the era of advanced technology and artificial intelligence: an integrative review. BMC Med Educ.

[3] Ang CS (2025). Developing AI literacy in healthcare education: bridging the gap in competency assessment. Discov Educ.

[4] Gazquez-Garcia J, Sánchez-Bocanegra CL, Sevillano JL (2025). AI in the health sector: systematic review of key skills for future health professionals. JMIR Med Educ.

[5] Getenet S, Cantle R, Redmond P, Albion P (2024). Students’ digital technology attitude, literacy and self-efficacy and their effect on online learning engagement. Int J Educ Technol High Educ.

[6] Abou Hashish EA, Alnajjar H (2024). Digital proficiency: assessing knowledge, attitudes, and skills in digital transformation, health literacy, and artificial intelligence among university nursing students. BMC Med Educ.

[7] Brod C (1984). Technostress: the human cost of the computer revolution.

[8] Ragu-Nathan TS, Tarafdar M, Ragu-Nathan BS, Tu Q (2008). The consequences of technostress for end users in organizations: conceptual development and empirical validation. Inf Syst Res.

[9] Califf CB, Sarker S, Sarker S (2020). The bright and dark sides of technostress: a mixed-methods study involving healthcare IT. MIS Q.

[10] Ibrahim RK, Al Marar YA, Salman M, Jehad S, Hamza MG, Abouelnasr AS (2025). Impact of multiple educational technologies on well-being: the mediating role of digital cognitive load. BMC Nurs.

[11] Arvai N, Katonai G, Mesko B (2025). Health Care Professionals’ concerns about medical AI and psychological barriers and strategies for successful implementation: scoping review. J Med Internet Res.

[12] Si J (2025). Exploring AI literacy, attitudes toward AI, and intentions to use AI in clinical contexts among healthcare students in Korea: a cross-sectional study. BMC Med Educ.

[13] Amiri H, Peiravi S, Rezazadeh Shojaee SS, Rouhparvarzamin M, Nateghi MN, Etemadi MH (2024). Medical, dental, and nursing students’ attitudes and knowledge towards artificial intelligence: a systematic review and meta-analysis. BMC Med Educ.

[14] Carolus A, Koch MJ, Straka S, Latoschik ME, Wienrich C (2023). MAILS: meta AI literacy scale: development and testing of an AI literacy questionnaire based on well-founded competency models and psychological change-and meta-competencies. Comput Hum Behav Artif Hum.

[15] Tarafdar M, Tu Q, Ragu-Nathan BS, Ragu-Nathan TS (2007). The impact of technostress on role stress and productivity. J Manag Inf Syst.

[16] Cabı E (2016). Attitude scale for digital technology. Kastamonu Educ J.

[17] Field A (2013). Discovering statistics using IBM SPSS Statistics.

[18] Kline RB (2015). Principles and practice of structural equation modeling.

[19] Cheung GW, Rensvold RB (2002). Evaluating goodness-of-fit indexes for testing measurement invariance. Struct Equ Model.

[20] Bentler PM (1990). Comparative fit indexes in structural models. Psychol Bull.

[21] Browne MW, Cudeck R, Bollen KA, Long JS (1993). Testing structural equation models.

[22] Kimiafar K, Sarbaz M, Tabatabaei SM, Ghaddaripouri K, Mousavi AS, Mehneh MR (2023). Artificial intelligence literacy among healthcare professionals and students: a systematic review. Front Health Inf.

[23] Özbek Güven G, Yilmaz Ş, Inceoğlu F (2024). Determining medical students’ anxiety and readiness levels about artificial intelligence. Heliyon.

[24] Kumpikaitė-Valiūnienė V, Aslan I, Duobienė J, Glińska E, Anandkumar V (2021). Influence of digital competence on perceived stress, burnout and well-being among students studying online during the COVID-19 lockdown: a 4-country perspective. Psychol Res Behav Manag.

[25] Laupichler MC, Aster A, Meyerheim M, Raupach T, Mergen M (2024). Medical students’ AI literacy and attitudes towards AI: a cross-sectional two-center study using pre-validated assessment instruments. BMC Med Educ.

[26] Cho KA, Seo YH (2024). Dual mediating effects of anxiety to use and acceptance attitude of artificial intelligence technology on the relationship between nursing students’ perception of and intention to use them: a descriptive study. BMC Nurs.

[27] Subaveerapandiyan A, Mvula D, Ahmad N, Taj A, Ahmed MG (2024). Assessing AI literacy and attitudes among medical students: implications for integration into healthcare practice. J Health Organ Manag.

[28] Tarafdar M, Cooper CL, Stich JF (2019). The technostress trifecta: techno eustress, techno distress and design: theoretical directions and an agenda for research. Inf Syst J.

[29] Mushtaque I, Awais-E-Yazdan M, Waqas H (2022). Technostress and medical students’ intention to use online learning during the COVID-19 pandemic in Pakistan: the moderating effect of computer self-efficacy. Cogent Educ.

[30] Liu CF, Cheng TJ, Chen CT (2019). Exploring the factors that influence physician technostress from using mobile electronic medical records. Inform Health Soc Care.

[31] Keshavarz H, Saeidnia HR, Wang T (2025). Navigating technostress: a deep dive into health practitioners’ technological challenges in hospital settings. BMC Health Serv Res.

[32] Ibrahim RK, Al Sabbah S, Al-Jarrah M, Senior J, Almomani JA, Darwish A (2024). The mediating effect of digital literacy and self-regulation on the relationship between emotional intelligence and academic stress among university students: a cross-sectional study. BMC Med Educ.

[33] Vermisli S, Cevik E, Cevik C (2022). The effect of perceived stress and digital literacy on student satisfaction with distance education. Rev Esc Enferm USP.

[34] Ayed A, Ejheisheh MA, Al-Amer R, Aqtam I, Ali AM, Othman EH (2025). Insights into the relationship between anxiety and attitudes toward artificial intelligence among nursing students. BMC Nurs.

[35] Golz C, Peter KA, Müller TJ, Mutschler J, Zwakhalen SM, Hahn S (2021). Technostress and digital competence among health professionals in Swiss psychiatric hospitals: cross-sectional study. JMIR Ment Health.

[36] Lugassy D, Levanon Y, Pilo R, Shelly A, Rosen G, Meirowitz A (2018). Predicting the clinical performance of dental students with a manual dexterity test. PLoS One.

[37] Inamdar MN, Munaga S, Khare N, Farooq MU (2020). Development of psychomotor skills in dentistry based on motor learning principles: a review. World J Dent.

[38] Blatz MB, Chiche G, Bahat O, Roblee R, Coachman C, Heymann HO (2019). Evolution of aesthetic dentistry. J Dent Res.

[39] Caplin RL (2021). Dentistry: art or science?: has the clinical freedom of the dental professional been undermined by guidelines, authoritative guidance and expert opinion?. Br Dent J.

[40] Kwon JH, Shuler CF, von Bergmann H (2022). Professional identity formation: the key contributors and dental students’ concerns. J Dent Educ.

[41] Du X, Al Khabuli JO, Ba Hattab RA, Daud A, Philip NI, Anweigi L (2023). Development of professional identity among dental students: a qualitative study. J Dent Educ.

[42] Jeong H, Han SS, Jung HI, Lee W, Jeon KJ (2024). Perceptions and attitudes of dental students and dentists in South Korea toward artificial intelligence: a subgroup analysis based on professional seniority. BMC Med Educ.

